# Comparison of Ferguson’s *δ* and the Gini coefficient used for measuring the inequality of data related to health quality of life outcomes

**DOI:** 10.1186/s12955-020-01356-6

**Published:** 2020-04-28

**Authors:** Hsien-Yi Wang, Willy Chou, Yang Shao, Tsair-Wei Chien

**Affiliations:** 1 Department of Nephrology, Chi-Mei Medical Center, Tainan, Taiwan; 2 Department of Sport Management, College of Leisure and Recreation Management, Chia Nan University of Pharmacy and Science, Tainan, Taiwan; 3 Department of Physical Medicine and Rehabilitation, Chung Shan Medical University, Taichung, Taiwan; 4 Department of Physical Medicine and Rehabilitation, Chiali Chi Mei Hospital, Tainan, Taiwan; 5 School of Fashion and Design, Jiaxing Vocational and Technical College, Jiaxing, China; 6 Departments of Medical Research, Chi-Mei Medical Center, 901 Chung Hwa Road, Yung Kung Dist., Tainan, 710 Taiwan

**Keywords:** Ferguson’s *δ*, Gini coefficient, Cronbach’s *α*, Dimension coefficient, Quality of life

## Abstract

**Background:**

Ferguson’s *δ* and Gini coefficient (GC) are defined as contrasting statistical measures of inequality among members within populations. However, the association and cutting points for these two statistics are still unclear; a visual display is required to inspect their similarities and differences.

**Methods:**

A simulation study was conducted to illustrate the pertinent properties of these statistics, along with Cronbach’s *α* and dimension coefficient (DC) to assess inequality. We manipulated datasets containing four item lengths with two number combinations (0 and 33%) in item length if two domains exist. Each item difficulty with five-point polytomous responses was uniformly distributed across a ± 2 logit range. A simulated response questionnaire was designed along with known different structures of true person scores under Rasch model conditions. This was done for 20 normally distributed sample sizes. A total of 320 scenarios were administered. Four coefficients (Ferguson’s *δ*, GC, test reliability Cronbach’s *α*, and DC) were simultaneously calculated for each simulation dataset. Box plots were drawn to examine which of these presented the correct property of inequality on data. Two examples were illustrated to present the index on Google Maps for securing the discriminatory power of individuals.

**Results:**

We found that 1-Ferguson’s δ coefficient has a high correlation (0.95) with GC. The cutting points of Ferguson’s *δ*, GC, test reliability Cronbach’s *α*, and the DC are 0.15, 0.50, 0.70, and 0.67, respectively. Two applications are shown on Google Maps with GCs of 0.14 and 0.42, respectively. Histogram legends and Lorenz curves are used to display the results.

**Conclusion:**

The GC is recommended to readers as an index for measuring the extent of inequality (or lower discrimination power) in a given dataset. It can also show the study results of person measures to determine the inequality in the health-related quality of life outcomes.

## Background

The required measurement properties of health-related quality of life (QoL) questionnaires are reliability, discriminatory power, and validity. Traditionally, assessment measurements are formally evaluated using the indices of reliability (the degree of measurement error) and validity (the extent to which the questionnaire measures what it is supposed to measure) [1].

Ferguson’s *δ* [2] was applied in studies published before 2007 to measure the discriminatory power of a test [3, 4]. Hankins provided a generalized formula for calculating Ferguson’s δ for questionnaires with dichotomous and polytomous items [1] and then (re-)introduced the coefficient δ as an index of discrimination to be distinguished from the well-known validity and reliability measurement properties [5, 6].

However, Hankins’ paper [1] resulted in critical comments from Wyrwich [7] and Norman [8] regarding reliability issues. Hankins [1] (1) applied Ferguson’s *δ* to identify the discrimination of GHQ-12 data using the dichotomous scoring method and four-point Likert-type scoring method, and (2) showed that the Likert-type scoring method could better discriminate between individuals compared with the dichotomous method. Moreover, as expected, the four-point Likert scale showed higher reliability than the dichotomous method. Hankins responded to the comments [9] by stating that, aside from reliability and validity, Ferguson’s δ is an additional index of an instrument’s measurement properties, i.e., Ferguson’s δ can only be computed on the assumption that the measurement is valid and reliable [9]. Terluin and his colleagues [6] stated that the magnitude of Ferguson’s δ is only determined by the distribution of the scores in a given sample. They also argued that Ferguson’s δ is an unnecessary property measure because it ignores reliability, making it impossible to interpret when questionnaire reliability is considered [6].

Despite these discussions [1, 5–9] in 2007 and 2008, several papers [10–12] were published, in which Ferguson’s δ was used to measure the scale discriminatory power between individuals. In Eq. (1), we list the traditional Ferguson’s *δ* used for the binary scale and polytomous items developed by Hankins [1]. The original Ferguson’s formula is simplified to the Guilford’s equation (i.e., the 2nd part in Eq. (1)) [13] and developed in line with Hankins’ formula (i.e., the 3rd part used for the polytomous scale) given by [1]

1 $$ {\displaystyle \begin{array}{l}\delta =\frac{n^2-\sum \limits_{i=0}^k{f}_i^2}{n^2-\frac{n^2}{\left(k+1\right)}}=\frac{\left(k+1\right)\left({n}^2-\sum \limits_{i=0}^k{f}_i^2\right)}{k\left({n}^2\right)}=\frac{\left(k\left(m-1\right)+1\right)\left({n}^2-\sum \limits_{i=0}^{k\left(m-1\right)}{f}_i^2\right)}{k\left(m-1\right)\left({n}^2\right)},\\ {}\end{array}} $$

where *n* is the number of elements (or summation of all frequencies), *f* is the frequency of score *i*, *k* is the number of questionnaire items, and *m* is the length of the scale (i.e., number of the threshold for a rating scale). Terluin et al. [6] proposed a standard computation of Ferguson’s *δ* (see Eq. (2)) expressed as

2 $$ \delta =\frac{q}{q-1}\times \frac{\left({n}^2-\sum \limits_{i=1}^q{f}_i^2\right)}{\left({n}^2\right)}=\frac{q}{q-1}\times \left(1-\frac{\sum \limits_{i=1}^q{f}_i^2}{\left({n}^2\right)}\right)=\frac{q}{q-1}\left(1-\sum \limits_{i=1}^q\frac{f_i^2}{n^2}\right)=\frac{q}{q-1}\left(1-\sum \limits_{i=1}^q{p}_i^2\right), $$

where *q* represents the possible score categories (i.e., number of bins for all elements) and *p* is the proportion for each frequency to the total number of persons. Referring to the reliability, commonly represented by Cronbach’s *α* [14] and shown in Eq. (3), where *K* is the item length, we can see that Eq. (3) is very similar to Eq. (2), particularly in the 2nd part.

3 $$ \alpha =\frac{k}{k-1}\left(1-\frac{\sum \limits_{i=1}^I{\sigma}_i^2}{\sigma_x^2}\right) $$

The difference lies in the numerator, which is the sum of identical elements across all bins in Eq. (2) and the summed variances across items in Eq. (3). As can be seen, the property of Ferguson’s *δ* is almost involved in Cronbach’s *α* if we reverse *δ* as (1 − *δ)*.

Furthermore, the Gini coefficient (GC) [15] is a measure of statistical dispersion to represent the income or wealth distribution of a nation’s residents (see Eq. (4)) and is expressed as.

4 $$ \mathrm{Gini}=\frac{q}{q-1}\times \frac{\sum \limits_i\sum \limits_j\mid {X}_i-{X}_j\mid }{2\sum \limits_i\sum \limits_j\overline {X_{ij}}}=\frac{q}{q-1}\times \frac{\sum \limits_i\sum \limits_j\mid {X}_i-{X}_j\mid }{2\times {q}^2\times {\overline{X}}_{ij}}, $$

where *X* is the frequency for each element, *X-bar* refers to all elements in frequencies or bins, and *q* is the number of bins. The numerator denotes the total absolute deviation between frequencies in bins, and the denominator represents the maximal portion of the total difference. We can also clearly see in Eq. (4) That the property of Ferguson’s *δ* is similar to GC if we reverse *δ* as (1 − *δ)*. Given that Eqs. (2)–(4) are very similar, whether they have high correlations is worthy of investigating. Thus, we aim to inspect the associations among the three indices, examine their validity and dimension coefficient (DC) [16], and verify whether GC can replace Ferguson’s *δ* as an index of discriminatory power between individuals.

The objectives of this study are as follows: to (i) compare the relationship between GC and Ferguson’s *δ*; (ii) to examine the associations among these four indices using simulation data; and (iii) to illustrate applications of the usefulness on GC in practice.

## Methods

### Simulative datasets

The simulated data contain four item lengths (i.e., 5, 10, 15, and 20) with two number combinations (i.e., 0 and 33%) in item length if two domains exist (e.g., 7 items on a domain and 3 on another when item length = 10).

Each item difficulty, with five-point polytomous responses, was uniformly distributed across a ± 2 logit range, and the questionnaire response was interacted by person ability and item difficulty under Rasch model conditions [17]. This was carried out for 20 normally distributed sample sizes (n from 50 to 1000 with an interval of 50). The detailed steps are stated below.

(A). The questionnaire responses were determined by (1) person ability and (2) item difficulties [17].
(B). A total of 320 simulation datasets were manipulated as follows: (1) four types of item lengths (i.e., 5, 10, 15, and 20), (2) four kinds of item loadings (i.e., 0.3, 0.5, 0.7, and 1.0) to the test domain, and (3) 20 normally distributed sample sizes (n from 50 to 1000 with an interval of 50).
(C). Based on the terms stated in (B) above, item difficulties on each type of item length were uniformly distributed across a ± 2 logit range, and the summation was equal to zero (i.e., the mean of all item difficulties = 0). For instance, five items had difficulties of {− 2, − 1, 0, 1, 2}. Other types of item length (e.g., 10, 15, 20) were similarly assigned with diffident difficulties from − 2 to 2. The total difficulties for each dataset were equal to zero.
(D). Item loading to the test domain refers to the correlation of responses between the specific item and the domain (≒summation across all items). If all items have a high correlation to the domain, the scale can be unidimensional and considered to have a high construct validity (e.g., the DC > 0.9 [16]).
(E). The way to generate responses in a one-dimensional (1D) scale (i.e., all items measuring a common character or attribute, such as leadership) is to set all responses on items with high correlation. The processes are shown herein.
(F). First, we determined the person latent trait called variable *T*. Assuming the standardized summation score across all items followed a normal distribution; we applied the function of random number generation in MS Excel to produce variable T.
  Next, the responses by the person on the item were determined by the new variable *T*1 according to the formula (= T*cos (angle) + W*sin (angle)), where variable *W* is generated by normally distributed random numbers, and the angle is defined by RADIANS (angle degree) using the MS Excel function.
(G). The 1D scale was formed by performing step (F) on all items when *T*1 = *T* (i.e., corr(*T*, *T*1) ≒ 1.0) and responses are determined by the item difficulty and the person’s ability on *T*1.
  The two-domain scale is yielded by *T* and *T*1, where *T*1 ≠ *T*, corr(*T*, *T*1) ≒ correlation (=0.3, 0.5, or 0.7), and the ratio of item length on two domains is 7:3. The responses are generated by the item difficulties and *T*i, respectively.
(H). Finally, Rasch data were simulated following the process described in reference [17].

**Fig. 1:**
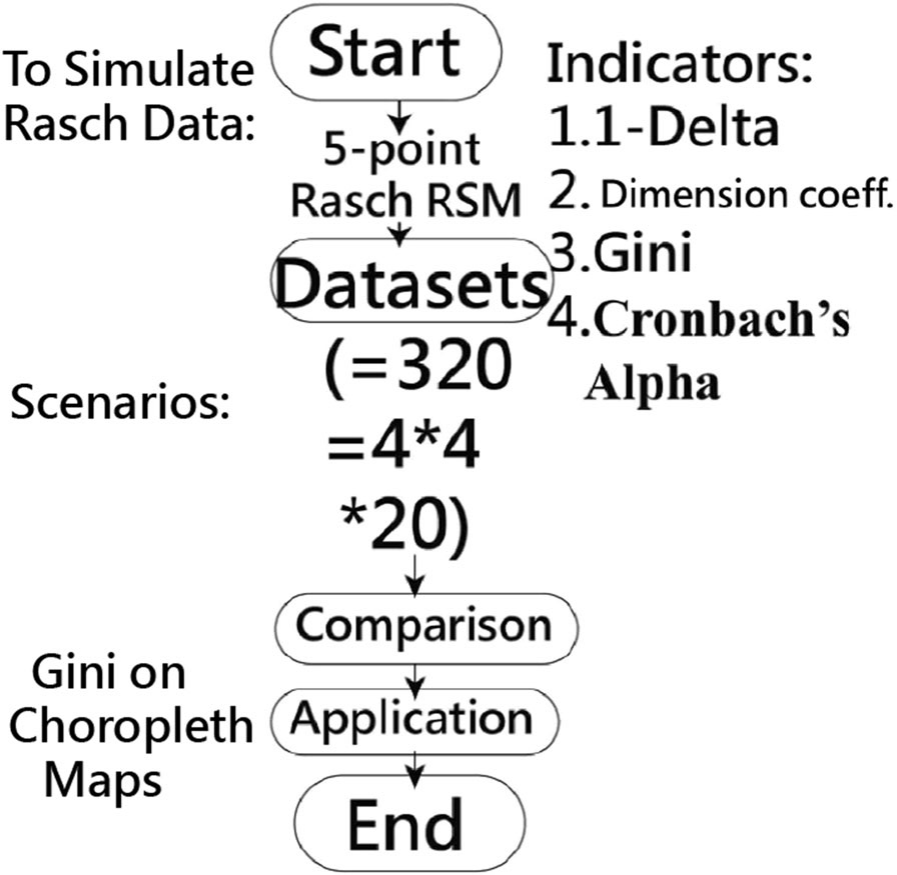
Study flowchart with data from 320 scenarios under the Rasch rating scale model

The four coefficients, Ferguson’s *δ*, GC, test reliability (Cronbach’s *α*), and DC, were simultaneously calculated for each simulation dataset (see Fig. 1). The simulation process for this study is presented in Additional file 1.

### Comparing the relationship between GC and Ferguson’s *δ*

Five sequential scores were designed as {1,2,3,4,5}, {1,2,3,4,5(10)}, {1,2,3,4,5(50)}, {1,2,3,4,5(300)}, and {1,2,3,4,5(900)}, where the numbers in parentheses denote the occurrence of the previous number; for instance, 5(10) means 5 occurs 10 times in the sequence. The results of (1 − Delta) and GC are expected to monotonously increase as the kurtoses are raised.

If we remove the adjustment of q/(q − 1) in Eq. (4), GC becomes 1, but only for a large population in which one person has all the income. For the 5-element set, where 4 has no income, and the fifth has all the income, the GC is 0.8. Thus, we adjusted Eq. (4) with the argument of q/(q − 1) ahead at the equation.

Next, we mimicked the World Bank’s method of calculating global wealth inequality for each county/area and divided resident incomes into five strata (i.e., lowest fifth, second fifth, third fifth, fourth fifth, and highest fifth). As a result, we were able to compare GCs with one another on a common base of five strata with equal size.

### Examining the associations among the four indices using simulation data

Ferguson’s *δ*, GC, Cronbach’s *α*, and DC were all examined using simulation data. Box plots were drawn to examine the property of inequality for the former two compared with the reliability and validity of the latter two. The cutting points of these four indices were also determined in this study.

### Illustrating the practical applications of GC

Two examples were illustrated to present GC on Google Maps for securing the discriminatory power of individuals.

(i) Liking for Science Questionnaire - This measures the attitudes of children to science-related activities [18]. It is an attitude survey with Likert scale ratings, where 0 = Dislike, 1 = Don’t know, and 2 = Like [18, 19]. The frequency values of examinees across bins on histogram were used to compute the GCs.
(ii) International author collaborations found in published papers on health-related QoL outcomes. After searching abstracts from MEDLINE with the keywords “Health Qual Life Outcomes” [Journal], a total of 2183 research articles were downloaded. These were then plotted on Google Maps using choropleth maps and Lorenz curves [20] to display the distribution of publication outputs across countries/areas for first authors. The frequency publication outputs of members across the world were used to compute GC using the quantile classification method (i.e., equal sizes in each class).

## Results

### Comparing the relationship between GC and Ferguson’s *δ*

The attributes of GC and (1-Delta) are shown in Table 1, which indicates close relations between the two coefficients. The results of (1 − Delta coefficient) and GC monotonously increase as the kurtoses are raised across scenarios (Table 1). The higher the (1 − Delta) or GC, the lesser the discriminatory power of individuals, thus indicating that unequal inequality exists.

**Table 1:** Comparison of (1-Delta) and Gini coefficient

Coefficient	Scenario A	Scenario B	Scenario C	Scenario D	Scenario E
data	1,2,3,4,5	1,2,3,4,5(10)	1,2,3,4,5(50)	1,2,3,4,5(300)	1,2,3,4,5(900)
n	5	14	54	304	904
Frequency	1,1,1,1,1	1,1,1,1,10	1,1,1,1,50	1,1,1,1300	1,1,1,1900
q	5	5	5	5	5
1-Delta	0	0.41	0.82	0.97	0.99
Gini	0	0.64	0.90	0.98	0.99

Note. Data are the summation scores for individuals; n = number of persons; Frequency = occurrence counts for each identical score; q = number of bins

Whether the higher GC (or 1-Delta) for person measures is negatively related to lower Cronbach’s *α* will be examined in the next section.

### Examining the association among the four indices using simulation data

**Fig. 2:**
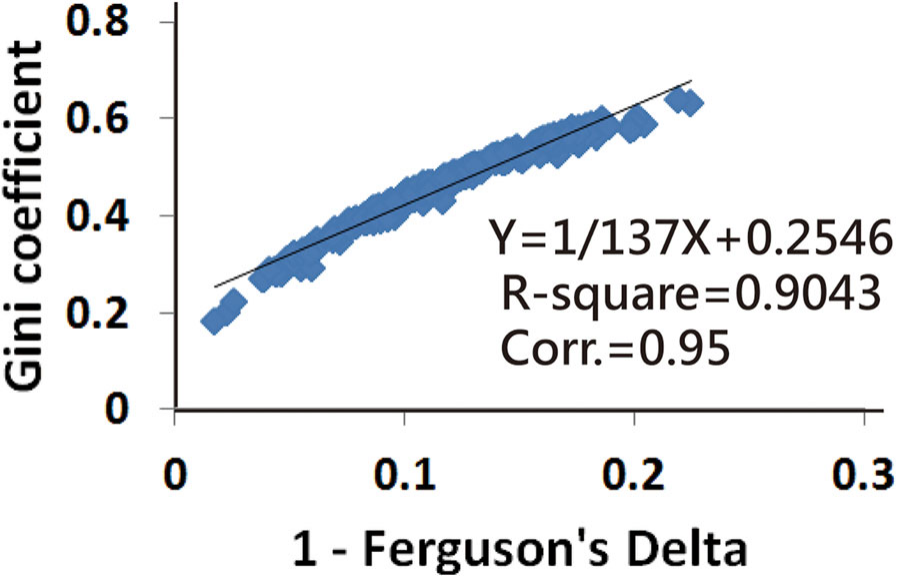
The relation between these two coefficients

**Fig. 3:**
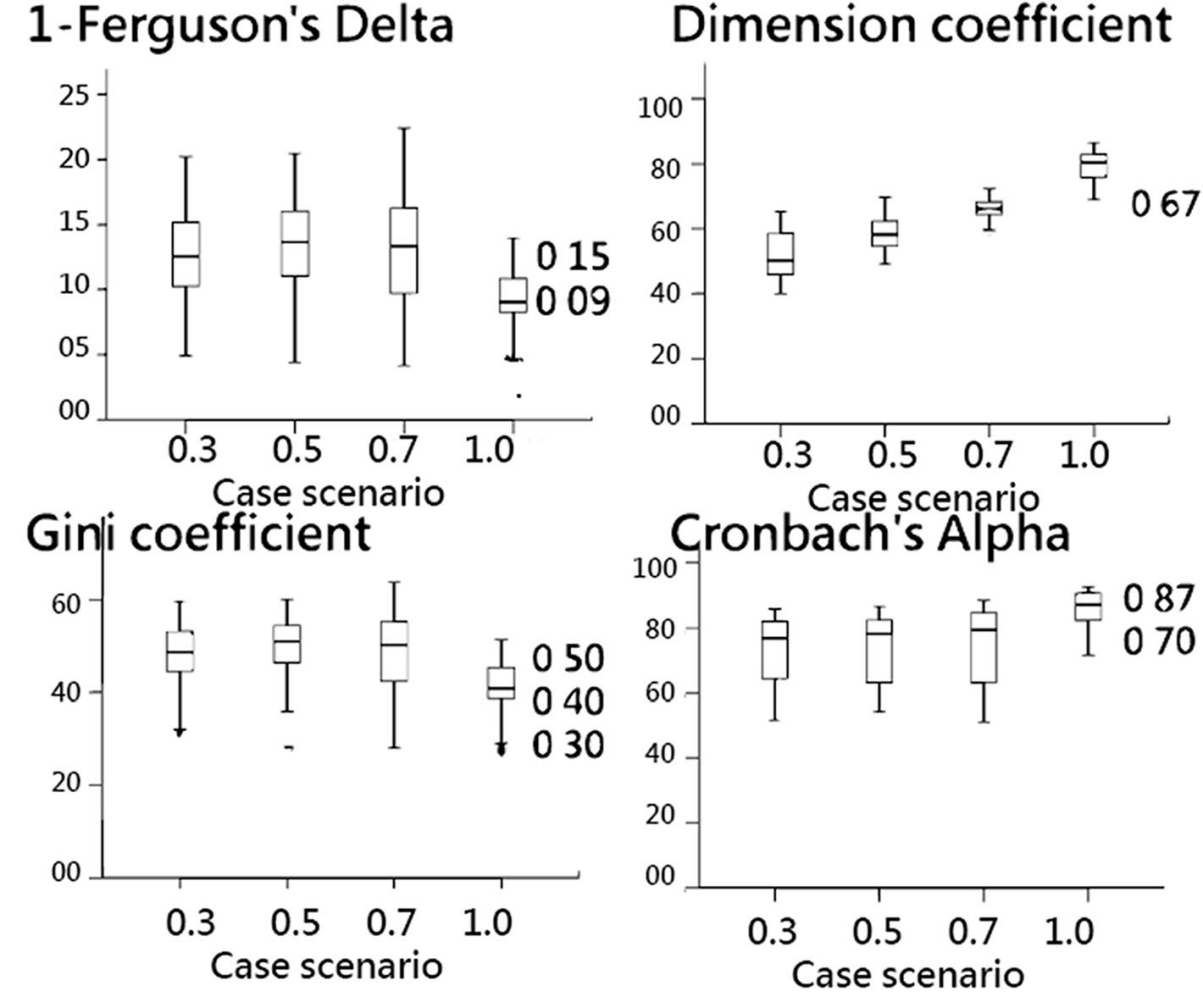
Comparisons of data distribution for the three study indicators

The correlation coefficient relation between the (1 − Delta) and GC indices is 0.95, and R-square = 0.90 (Fig. 2). The box plots in Fig. 3 show that (1 − Delta) and GC are closely related in contrast to Cronbach’s *α* and DC, which have a negative correlation, that is, the higher the Cronbach’s *α* (or DC), the greater the discriminatory power between individuals. In contrast, the higher the GC and (1-Delta), the lesser the discriminatory power between individuals [1, 5].

The DC is more sensitive to misfit items(i.e., with lower loadings to the test domain) than Cronbach’s *α*, thus implying that reliability is a necessary, but not sufficient, a component of validity [21, 22]. The DC is, therefore, necessarily incorporated with Cronbach’s α to completely and fully describe a scale’s characteristics [23]. This is because not all reliable scales are valid [24].

The cutting points are determined at 0.15 for (1 − Delta) or 0.85 for Delta, 0.50 for GC, 0.67 for DC, and 0.70 for Cronbach’s *α*. Particularly, we refer the common cutting point for Cronbach’s *α* at the lower limit (0.7) to the upper limit (0.5) for GC in Fig. 3. The result of the GC cutting point setting at 0.5 is similar to the previous study [25, 26].

### Illustrating the practical applications of using Gini coefficients

**Fig. 4:**
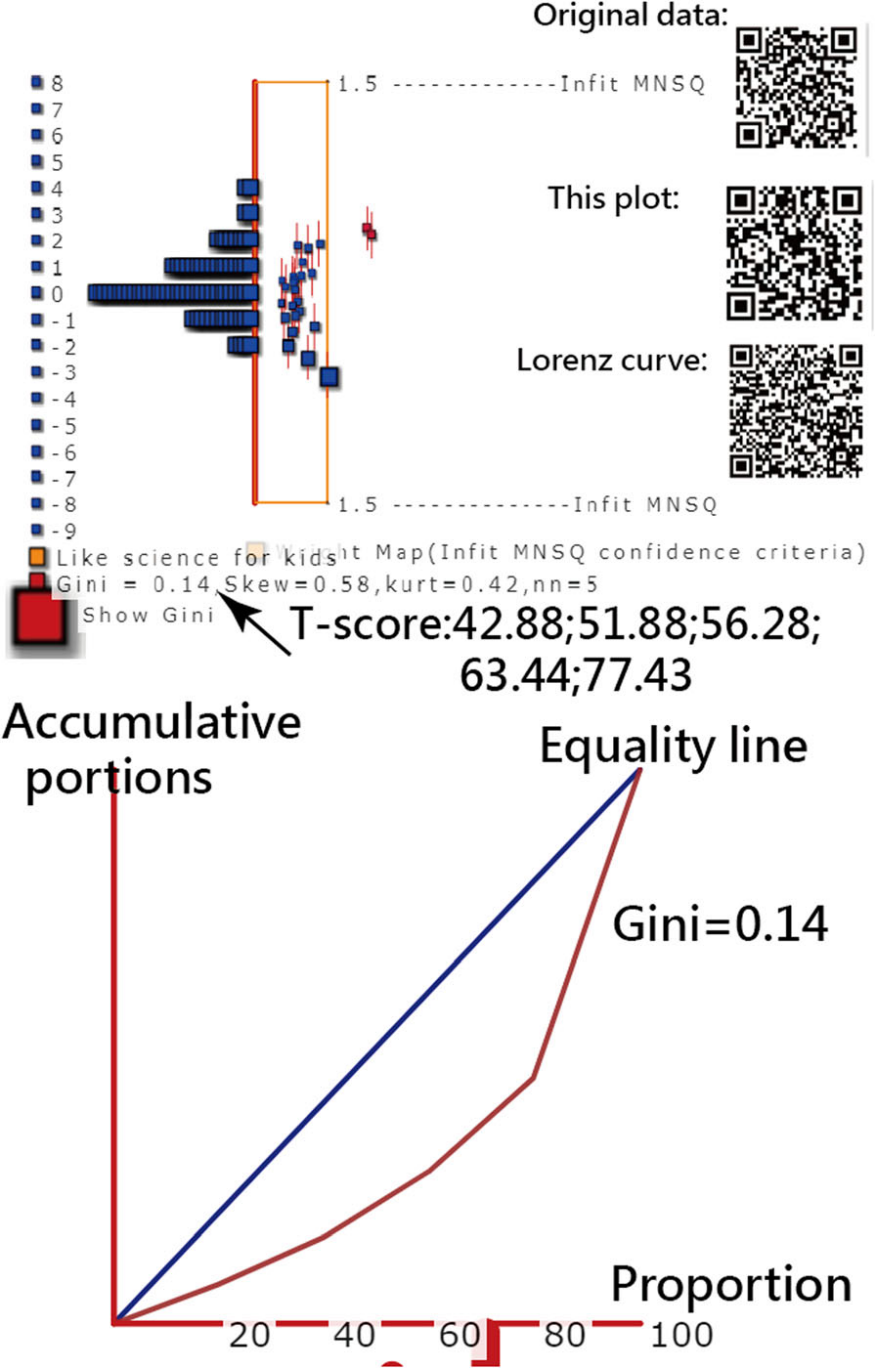
Gini coefficient sfor histogram shown on Google Maps

**Fig. 5:**
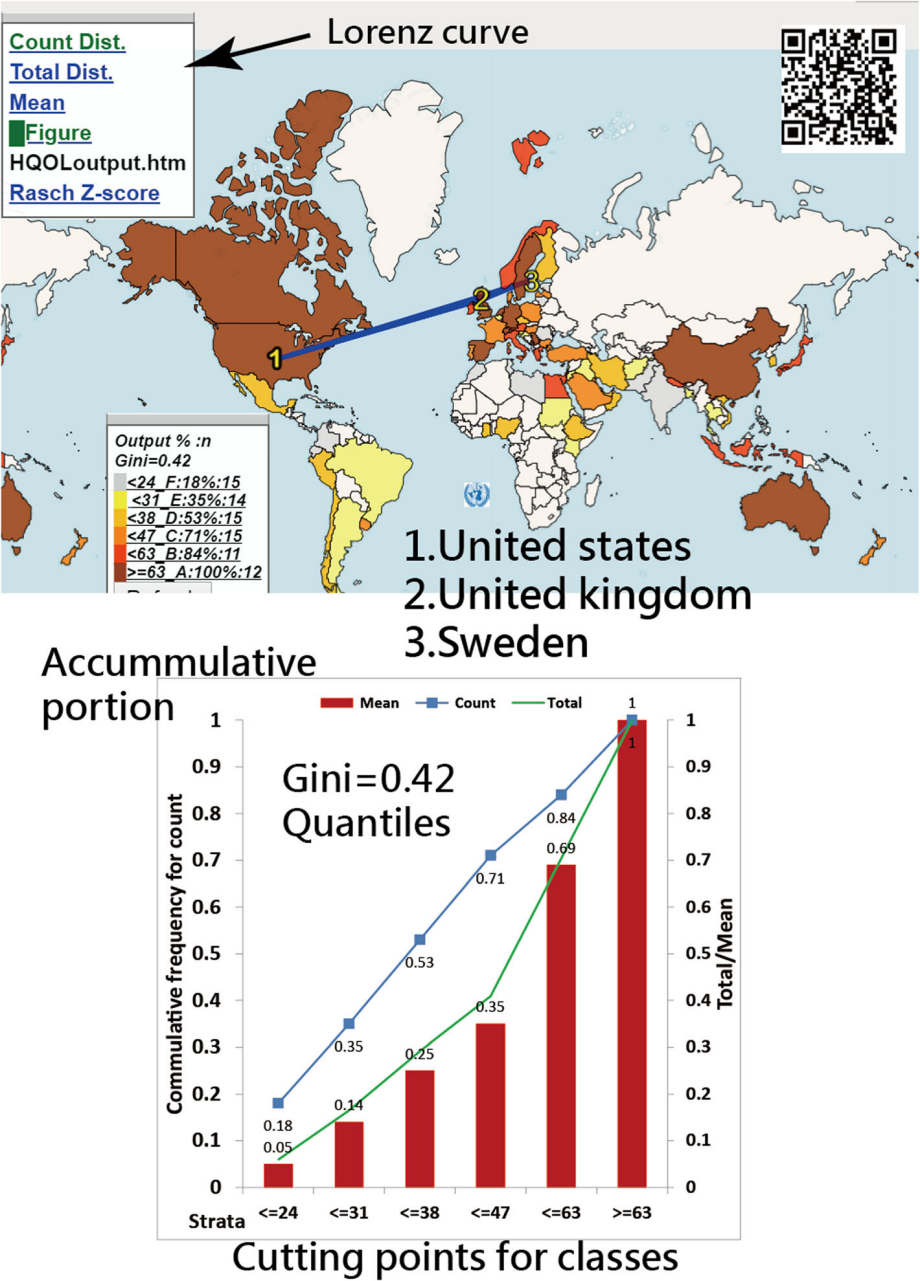
Distribution of countries/areas for author publications on Google Maps (GC = 0.14 on the top 5 clusters)

In Figs. 4 and 5, the GCs for the two applicable examples are shown on Google Maps [27, 28], where GCs = 0.14 and 0.42, respectively, indicating that the publication outputs based on countries/areas for Health Qual Life Outcomes and the individual performances for the Liking for Science Questionnaire present acceptable GCs (< 0.5).

However, the Cronbach’s *α* and DC values for the questionnaire in Fig. 4 are 0.88 and 0.64, respectively, indicating that the GC is negatively related to Cronbach’s *α*. Two items (Nos. 5 and 23) are a misfit to the Rasch model and lead to a lower DC (0.64 < 0.67) [16].

The top three most productive countries for Health Qual Life Outcome are the United States (224, 5.59%), the United Kingdom (205, 5.11%), and Sweden (200, 4.99%), respectively. Interested readers are invited to scan the Quick Response codes in Figs. 4 and 5 to see more details about the two practical examples.

## Discussion

This study finds that (i) GC can replace Ferguson’s *δ* as an additional index of an instruments measurement properties aside from reliability and validity, and (2) the method used by the World Bank to calculate the GC for each country/area, in which sample scores are divided into five strata with equal size, is practically feasible.

### What this adds to existing knowledge and what is known

Although Terluin et al. [6] argued that Ferguson’s δ becomes unnecessary when reliability is considered, Ferguson’s δ (or GC) can be an additional index aside from reliability and validity [9, 29]. In particular, GC at a cutting point of 0.5 is easier to use than Delta and can better determine a scale with effective discriminatory power.

For a long time, the alarming level of GC has been globally taken as 0.4, especially in World Bank calculations for global wealth inequality [25]. Although the 0.4 standards is widely accepted [26], the derivation of the value lacks rigid theoretical foundations. Our computations using simulation data show that the alarming level should be specified to be equal or larger than 0.5 based on the 95% confidence intervals rather than 0.4 at the median to the distribution. The result is similar to that presented by a previous study [25].

A normal distribution is expected to have a discrimination of Delta > 0.90 [1, 2], which is near the findings (1 − Delta = 0.09 or Delta ≥0.91) in Fig. 3. However, the lower boundary of Delta at 0.85 can provide readers an objective way to examine the discriminatory power, particularly for data following a uniform distribution. Many teachers are concerned about whether the abilities of students are equal. This is because the more equal the abilities of students, the more willing many teachers are to teach the class [30]. The GC can be used to compare the degree of equality between the academic abilities of students.

### What this implies and what should be changed

Our findings in Task 2 (i.e., Comparing the relationship between GC and Ferguson’s *δ*) corresponds with previous studies [16, 31], which suggest incorporating Cronbach’s α with DC or exploratory factor analysis to jointly assess scale quality. We see in Fig. 3 that the DC can discriminate scale dimension tendency more sensitively than Cronbach’s *α*_._ We also confirm that the cutting points are 0.7 for Cronbach’s *α* [32] and 0.67 for DC [16, 33].

In Task 3 (i.e., Illustrating practical applications of the usefulness of GC), we show visual displays on Google Maps, which enable users to gain an overall geospatial visualization [34, 35]. The GC across bins on the histogram (Figs. 4 and 5) can be an additional index shown to readers. Interested readers are recommended to use the methods shown in Additional files from 2 to 4 and Ref. [36] to easily compute the GCs on their own.

### The strengths of this study

Due to authors in previous five articles [5–9] discussing the value of Ferguson’s *δ* in 2007 and 2008, we complemented these article in details about the feature of Ferguson’s *δ* related to other coefficients(e.g., Gini coefficient and Cronbach’s Alpha), including Equations from (1) to (4) and verifications of Ferguson’s *δ* that *can be replaced with G*ini coefficient for measuring the extent of inequality (or lower discrimination power) in a given dataset based on a quality-of-care scale.

Furthermore, we applied legends along with choropleth maps [37, 38] to report GCs (< 0.50) and to ensure a higher discriminatory power (or say equal sizes in classes) on the Questionnaire (in Task 3, Fig. 4) and the international coauthor collaborations in papers extracted using the search term “Health Qual Life Outcomes” (Fig. 5) [28]. To the best of our knowledge, this is a distinct application that has never been used in previous papers.

We also developed a visual display to present the survey results in Ref. [26], that is, we presented the histograms and the GCs on Google Maps based on cloud computation. The way we incorporated choropleth maps and legends with Google Maps is a unique approach compared with other research methods [39–41]. This is because we used the dashboard to present the study results, which are better displayed than in traditional image formats. Interested readers can even manipulate the links according to their methods to understand the features of interest, such as the examinee distribution [26] and the international coauthor collaboration [27]. As the saying goes, “A picture is worth a thousand words,” and about this, we hope that future studies can report on other types of information using Google’s application programming interface.

## Limitations and directions for future study

Our study has some limitations. First, only 320 simulation scenarios were conducted(i.e., equal to 4 item length × 4 numbers of misfit items × 20 sample sizes). Hence, caution should be exercised when using the inference of this study, as many other scenarios in the real world have not been included.

Second, the GC calculation followed the World Bank’s GC calculation for a country/area, in which incomes are divided into five strata. For this reason, the feasibility and applicability should be further proved in the future even though any kind of data (i.e., count or continuous variables) can be easily applied to compute GC and objectively compared to others because all values are based on an equal number of observations in bins.

Third, the data in Task 3 were extracted from the MEDLINE Library and were carefully addressed. Every linkage was examined as correctly as possible. The originally downloaded contexts included some errors in symbols that might affect the resulting reports in this study, such as those shown in Fig. 5.

Fourth, the simulation data were processed under Rasch model conditions. The results in this study, such as the determination of cutting points for each index (Fig. 3) might be different from those of the other situations. For instance, a single variable is generated by a normal or uniform distribution or based on other types of item response theory. Hence, future studies regarding the determination of cutting points for indices are encouraged, and other conditions should be used to simulate data in the future.

## Conclusion

The GC is recommended to readers as an index to measure the extent of inequality (or lower discrimination power) in a given dataset. The GCs can also show the study results of person measures to determine the inequality in health-related QoL outcomes.

## Supplementary information

**Additional file 1.** The process of simulation data in this study at https://youtu.be/5BLJtiif2M4.

**Additional file 2.** MP4 showing the simulation process http://www.healthup.org.tw/marketing/course/marketing/raschsimulateddata.mp4.

**Additional file 3.** MS Word for showing the codes of simulation.

**Additional file 4.** Excel file to calculate Delta and GC as well as contents for this study.

## Data Availability

All items and data can be obtained from those in additional supporting files of this study.

## Abbreviations

1D: one dimension
CC: correlation coefficient
DC: dimension coefficient
EFA: exploratory factor analysis
GC: Gini coefficient
HQLO: Health Quality of Life Outcomes
IRT: item response theory
SNA: social network analysis
