# Supplementary material for: Comparison of Ferguson’s δ and the Gini coefficient used for measuring the inequality of data related to health quality of life outcomes
Source: Health Qual Life Outcomes. 2020 Apr 28;18:111. doi: 10.1186/s12955-020-01356-6 (PMC7189694; doi:10.1186/s12955-020-01356-6)
Supplement: Supplementary file 3 — Additional file 3. MS Word for showing the codes of simulation. [file 12955_2020_1356_MOESM3_ESM.docx]

**Sub simulate()**

Application.ScreenUpdating = False

lastRow = Sheets("report").Cells(Sheets("report").Rows.Count, "B").End(xlUp).Row + 1

mrange = "A2:N" & lastRow

' Sheets("report").Range(mrange).ClearContents

lastRow = Sheets("simulation").Cells(Sheets("simulation").Rows.Count, "B").End(xlUp).Row

Lastcol = Sheets("simulation").Cells(1, Sheets("simulation").Columns.Count).End(xlToLeft).Column

mrange = "a1:" & Sheets("simulation").Cells(lastRow, Lastcol).Address(False, False)

Sheets("simulation").Range(mrange).ClearContents

Sheets("data2").Range("b2:d1001").ClearContents

For jcorr = 1 To 5  **’4 types**

If jcorr = 1 Then Sheets("main").Cells(9, 1) = 0.3

If jcorr = 2 Then Sheets("main").Cells(9, 1) = 0.5

If jcorr = 3 Then Sheets("main").Cells(9, 1) = 0.7

If jcorr = 4 Then Sheets("main").Cells(9, 1) = 0.9

If jcorr = 5 Then Sheets("main").Cells(9, 1) = 1#

For jperson = 3 To 21  **’20 types**

n = Sheets("eigen").Cells(jperson, 1)

' n = 100

Sheets("main").Cells(7, 1) = n

If jperson >= 3 Then

aaaa = 1

End If

Application.ScreenUpdating = True

For jitem = 2 To 5 '21  **’4 types**

p = Sheets("eigen").Cells(1, jitem)

Sheets("main").Cells(7, 2) = p

Application.ScreenUpdating = True

diff = (4 + 1) / p

For j = 2 To p + 1  **’setting item difficulties**

diff2 = diff * (j - 2) + -2 'give the item difficulty

Sheets("simulation").Cells(1, j) = diff2

Next j

Sheets("simulation").Cells(1, 1) = "=average(B1:CC1)"

mean = Sheets("simulation").Cells(1, 1).Value

For j = 2 To p + 1 **’setting the mean of item difficulties=0**

diff2 = Sheets("simulation").Cells(1, j) - mean

Sheets("simulation").Cells(1, j) = diff2

Next j

mrange = "F2:F" & n + 1 & ",H2:H" & n + 1

Sheets("simulation2").Cells(5, 10) = "=CORREL(" & mrange & ")"

Sheets("simulation2").Range("A2:A2000").ClearContents

Sheets("simulation2").Range("F2:G2000").ClearContents

For jk = 2 To n + 1  **’latent train scores**

Sheets("simulation").Cells(jk, 1) = gauss

Sheets("simulation2").Cells(jk, 7) = gauss ' referring to correlation ability2

Sheets("simulation2").Cells(jk, 6) = Sheets("simulation").Cells(jk, 1)

Next jk

kcorr = Sheets("main").Cells(9, 1)

For j = 90 To -90 Step -1

Sheets("simulation2").Cells(1, 10) = j

If Sheets("simulation2").Cells(5, 10) < kcorr + 0.02 And Sheets("simulation2").Cells(5, 10) > kcorr - 0.02 Then  **’obtaining equivalent correlations of variable**

corr2 = j

Exit For

End If

Next j

If kcorr < 1 Then  **’for two domains**

For jk = 2 To n + 1

Sheets("simulation2").Cells(jk, 1) = Sheets("simulation2").Cells(jk, 8)

Next jk

Else  **’for one-dimensional scale**

For jk = 2 To n + 1

Sheets("simulation2").Cells(jk, 1) = Sheets("simulation2").Cells(jk, 6)

Next jk

End If

Simulation_random  **’simulate Rasch data**

halfitem = Sheets("main").Cells(7, 3)

For jm2 = 2 To n + 1  **’sum scores for two domains**

mcell2 = Sheets("data2").Cells(jm2, p - halfitem + 1).Address(False, False)

mcell3 = Sheets("data2").Cells(jm2, p - halfitem + 2).Address(False, False)

mcell4 = Sheets("data2").Cells(jm2, p + 1).Address(False, False)

Sheets("data2").Cells(jm2, 1) = "=sum(data!b" & jm2 & ":" & mcell2 & ")"

Sheets("data2").Cells(jm2, 2) = "=sum(data!" & mcell3 & ":" & mcell4 & ")"

Next jm2

Sheets("sheet1").Cells(Sheets("main").Cells(7, 2) + 8, 6) = Sheets("main").Cells(7, 2)

Sheets("sheet1").Cells(Sheets("main").Cells(7, 2) + 8, 7) = jcorr

Sheets("sheet1").Cells(Sheets("main").Cells(7, 2) + 8, 8) = jperson

simu_2 **’to get DC and correlation matrix**

Call btnOK ‘**’to get eigen values and factor score**

lastRow = Sheets("report").Cells(Sheets("report").Rows.Count, "B").End(xlUp).Row + 1

Sheets("report").Cells(lastRow - 1, 26) = Sheets("data_p").Cells(1, 1)

Sheets("report").Cells(lastRow - 1, 25) = Sheets("data_p").Cells(1, 2)

Sheets("report").Cells(lastRow - 1, 23) = Sheets("data2").Cells(1, 2)

Sheets("report").Cells(lastRow - 1, 24) = Sheets("data2").Cells(1, 4)

Sheets("report").Cells(lastRow - 1, 21) = Sheets("main").Cells(10, 1)

Sheets("report").Cells(lastRow - 1, 22) = Sheets("main").Cells(10, 2)

**’save results to worksheets**

Next jitem

Next jperson

Next jcorr

Application.ScreenUpdating = True

**End Sub**
